# Supplementary material for: Epigenetic Age Acceleration in Mothers and Offspring 4–10 Years after a Pregnancy Complicated by Gestational Diabetes and Obesity
Source: Metabolites. 2022 Dec 7;12(12):1226. doi: 10.3390/metabo12121226 (PMC9785326; doi:10.3390/metabo12121226)
Supplement: Supplementary file 1 [file metabolites-12-01226-s001.zip › metabolites-2037191-supplementary.pdf]

Analysis: No adjustment for BMI

Supplementary Table. 1 Multivariable Linear Regression of the association between biomarkers with DNAm age acceleration residual in Mothers (N=137)

Variable	Horvath Age Acceleration		Hannum Age Acceleration	
	β (SE)	P-value	β (SE)	P-value
HDL (mg/dL)	-0.071(0.038)	0.0670	-0.083(0.036)	**0.0213**
Leptin	-0.006(0.013)	0.6657	-0.004(0.013)	0.7807
HOMA-IR	0.101(0.145)	0.4878	0.073(0.136)	0.5912
Fasting Glucose (mg/dL)	-0.002(0.012)	0.8567	-0.005(0.011)	0.6199
Fasting Insulin (μU/ml)	0.052(0.051)	0.3077	-0.386(0.537)	0.4349

*Adjusted for chronological age, plate, and cell-type (CD8T, CD4T, NK, Bcell, Mono, Gran)

Supplementary Table. 2 Multivariable Linear Regression of the association between biomarkers with DNAm age acceleration residual in Offspring (N=116)

Variable	Horvath Age Acceleration		Hannum Age Acceleration	
	β (SE)	P-value	β (SE)	P-value
HDL (mg/dL)	0.025(0.058)	0.6624	0.021(0.049)	0.6740
Leptin	-0.016(0.043)	0.6989	-0.015(0.036)	0.6801
HOMA-IR	0.3996(0.310)	0.2009	0.401(0.261)	0.1274
Fasting Glucose (mg/dL)	0.085(0.086)	0.3207	0.054(0.072)	0.4551
Fasting Insulin (μU/ml)	0.109(0.076)	0.1550	0.106(0.064)	0.0997

*Adjusted for chronological age, sex, plate, and cell-type (CD8T, CD4T, NK, Bcell, Mono, Gran)

Analysis: No adjustment for Cell Types

Supplementary Table. 3 Multivariable Linear Regression of the association between metabolites with DNAm age acceleration residual in Mothers (N=137)

Variable	Horvath Age Acceleration		Hannum Age Acceleration	
	β (SE)	P-value	β (SE)	P-value
HDL (mg/dL)	-0.043(0.049)	0.3725	-0.084(0.048)	0.0843
Leptin	-0.027(0.025)	0.2968	-0.014(0.025)	0.5781
HOMA-IR	-0.013(0.198)	0.9480	-0.047(0.198)	0.8139
Fasting Glucose (mg/dL)	0.003(0.015)	0.8403	-0.007(0.015)	0.6520
Fasting Insulin (μU/ml)	-0.002(0.0687)	0.9780	-0.001(0.069)	0.9849

*Adjusted for chronological age, current BMI, and plate

Supplementary Table. 4 Multivariable Linear Regression of the association between metabolites with DNAm age acceleration residuals in Offspring (N=116)

Variable	Horvath Age Acceleration		Hannum Age Acceleration	
	β (SE)	P-value	β (SE)	P-value
HDL (mg/dL)	0.043(0.066)	0.5132	0.005(0.064)	0.9356
Leptin	-0.056(0.061)	0.3646	-0.017(0.05965)	0.7796
HOMA-IR	0.575(0.367)	0.1195	0.678(0.353)	0.0576
Fasting Glucose (mg/dL)	0.029(0.096)	0.7615	-0.008(0.093)	0.9315
Fasting Insulin (μU/ml)	0.152(0.090)	0.0943	0.175(0.087)	**0.0465**

*Adjusted for chronological age, sex, BMI z-score, and plate

Analysis: Adjusted for GDM status

Supplementary Table. 5 Multivariable Linear Regression of the association between metabolites with DNAm age acceleration residual in Mothers (N=137)

Variable	Horvath Age Acceleration		Hannum Age Acceleration	
	β (SE)	P-value	β (SE)	P-value
HDL (mg/dL)	-0.063(0.044)	0.1507	-0.078(0.041)	0.0565
Leptin	-0.0257(0.022)	0.2429	-0.018(0.021)	0.3739
HOMA-IR	0.057(0.166)	0.7303	0.022(0.156)	0.8882
Fasting Glucose (mg/dL)	-0.005(0.013)	0.7076	-0.008(0.012)	0.4765
Fasting Insulin (μU/ml)	0.038(0.059)	0.5172	0.019(0.055)	0.7370

[*]Adjusted for chronological age, current BMI, GDM Status, and plate

Supplementary Table. 6 Multivariable Linear Regression of the association between metabolites with DNAm age acceleration residuals in Offspring (N=116)

Variable	Horvath Age Acceleration		Hannum Age Acceleration	
	β (SE)	P-value	β (SE)	P-value
HDL (mg/dL)	0.028(0.061)	0.6444	0.015(0.052)	0.7680
Leptin	-0.041(0.059)	0.4849	-0.012(0.049)	0.8054
HOMA-IR	0.461(0.343)	0.1817	0.533(0.287)	0.0657
Fasting Glucose (mg/dL)	0.086(0.088)	0.3297	0.062(0.074)	0.4052
Fasting Insulin (μU/ml)	0.128(0.0845)	0.1342	0.143(0.071)	**0.0459**

[*]Adjusted for chronological age, sex, BMI z-score, GDM Status, and plate
